# Optical Aptamer Probes of Fluorescent Imaging to Rapid Monitoring of Circulating Tumor Cell

**DOI:** 10.3390/s16111909

**Published:** 2016-11-23

**Authors:** Ji Yeon Hwang, Sang Tae Kim, Ho-Seong Han, Kyunggon Kim, Jin Soo Han

**Affiliations:** 1 Preclinical Research Center, Biomedical Research Institute, Seoul National University Bundang Hospital, 82, Gumi-ro 173beon-gil, Bundang-gu, Seongnam-si, Gyeonggi-do 13620, Korea; labanimal@snubh.org; 2 The Institute for the 3Rs, College of Veterinary Medicine, Konkuk University, 120 Neungdong-ro, Gwangjin-gu, Seoul 05029, Korea; 3 Department of Surgery, Seoul National University Bundang Hospital, 82, Gumi-ro 173beon-gil, Bundang-gu, Seongnam-si, Gyeonggi-do 13620, Korea; lingokst@snu.ac.kr (S.T.K.); hanhs@snubh.org (H.-S.H.); 4 Asan Institute for Life Sciences, Asan Medical Center, Seoul, Department of Medicine, University of Ulsan College of Medicine, 43 gil Olympic-ro, Pungnap dong, Songpa gu, Seoul 138-736, Korea; kimkyunggon@gmail.com

**Keywords:** EpCAM, aptamer, ALB, metastasis, molecular beacon

## Abstract

Fluorescence detecting of exogenous EpCAM (epithelial cell adhesion molecule) or muc1 (mucin1) expression correlated to cancer metastasis using nanoparticles provides pivotal information on CTC (circulating tumor cell) occurrence in a noninvasive tool. In this study, we study a new skill to detect extracellular EpCAM/muc1 using quantum dot-based aptamer beacon (QD-EpCAM/muc1 ALB (aptamer linker beacon). The QD-EpCAM/muc1 ALB was designed using QDs (quantum dots) and probe. The EpCAM/muc1-targeting aptamer contains a Ep-CAM/muc1 binding sequence and BHQ1 (black hole quencher 1) or BHQ2 (black hole quencher2). In the absence of target EpCAM/muc1, the QD-EpCAM/muc1 ALB forms a partial duplex loop-like aptamer beacon and remained in quenched state because the BHQ1/2 quenches the fluorescence signal-on of the QD-EpCAM/muc1 ALB. The binding of EpCAM/muc1 of CTC to the EpCAM/muc1 binding aptamer sequence of the EpCAM/muc1-targeting oligonucleotide triggered the dissociation of the BHQ1/2 quencher and subsequent signal-on of a green/red fluorescence signal. Furthermore, acute inflammation was stimulated by trigger such as caerulein in vivo, which resulted in increased fluorescent signal of the cy5.5-EpCAM/muc1 ALB during cancer metastasis due to exogenous expression of EpCAM/muc1 in Panc02-implanted mouse model.

## 1. Introduction

Tumor is the second leading cause of death worldwide. Therefore, the implementation of high activation and selective imaging modalities for timely tumor monitoring and tumor progression diagnosis are of great clinical importance. Particularly, circulating tumor cells (CTCs), tumor cells that have escaped from the tumor tissue mass, have the ability to disseminate metastatic sites and able to enter into the bloodstream, which may respond to tumor progression [1,2] However, the pathway for CTC dissociation and invasion into the bloodstream is a tangled process and is not yet fully understood due to the scarcity of detecting tools. There are only a very small number of CTCs in peripheral blood (within 1 CTC/10^7^ blood cells) [3]. Among the technologies that have been ensured to facilitate CTC monitoring from blood samples, most can be distinguished as those that target CTCs based on their surface antigens or on their physical size properties. Although morphological confirmation of CTC cells by their severe size, large nuclear profile, and cytoplasmic signature is readily available, they have tangled heterogeneous phenotypes. Once CTCs have been isolated, their monitoring is typically determined by immunocytochemical analysis, immunofluorescencing, or fluorescence microscopy analysis.

The expression level of cytokeratin, epithelial cell adhesion molecule (EpCAM), mucin1 (muc1) and lymphocyte common antigen (CD45) on CTCs can each vary. Recent studies have reported that several assays such as CTC and soluble tumor-related biomarkers may be present in the bloodstream even in the early stage of tumor progression [4]. However, the existence and expression of EpCAM or muc1 on CTC is not yet known due to the paucity of diagnostic tools. Some approaches have focused on detecting CTC directly from bloods by combination of several antibodies specific to unique tumor types, microfluidic system, reverse transcription-PCR and multiplex PCR [5,6,7,8,9]. However, it has been a huge challenge to detect these biomarkers with high sensitivity due to their infinitesimally concentration in the bloodstream [7]. The validity of CTC specific antibodies for in vivo applications is strictly hindered by several factors, such as their high immunogenicity, chemical modification and high production cost [10]. In addition, most of these approaches are limited by their slow speed and low CTC-detect yield. Certainly, accurate and sensitive detection of these target biomarkers is of great significance for timely disease prognosis and monitoring tumor metastasis.

Hence, one of the main issues in molecular imaging of CTC is the development of a detection method for infinitesimally concentration in the bloodstream. As small-sized oligonucleotides, aptamers are practically non-immunogenic and nontoxic in contrast to protein antibodies. Importantly, aptamers are also easily modified by integration of various functional parts, and aptamer-based analytical reagents can be easily stored or regenerated and reused. Aptamer sequences are designed via an in vitro selection process known as SELEX (systematic evolution of ligands by exponential enrichment), which entails a series of repetitive selection and amplification steps after interaction to the goal molecule [11]. In the past two decades, aptamers have been confirmed as a novel grade of nucleic acid-based molecular sensing probes, comprising short RNA or single-stranded DNA oligonucleotides (less than 75 bases in length) with unique stereoscopic structures that can recognize and bind to their targeting molecules with high specificity and affinity [12]. Aptamers offer many attractive benefits over protein antibodies that significantly promote their clinical application and effectiveness. This target-specific binding takes place through a conformational recognition process similar to the one mediating antibody-antigen reactions, and thus aptamers are often referred to as “chemical antibodies”. Importantly, aptamers recognize a various field of potential targets including chemical ions, drugs, DNA, RNA, peptides, proteins, viruses, live cells and tissues, and have the bioability to change conformation upon affinity to their selective targets. Some recent studies have demonstrated a core of monitoring tools using aptamers as sensitive recognition probes in integration with skills based on fluorescence, electrochemistry and others [13]. In another recent study, a cancer cell-activated fluorescent aptamer-reporter system was developed for sensitive detection of mucin 1, HER2 and estrogen receptor (ER) on CTCs [11,12,13,14,15,16,17]. Briefly, fluorophore and a paired quencher molecule were conjugated at the 5′-end and 3′-end of the aptamer sequences, respectively. In the absence of target molecules, the paired quencher molecules quenched the fluorescence. The reaction of the aptamer with molecules of interest, however, changed the sequence conformation, which induced a long distance of the fluorophore dye from the paired quencher molecule, and thus the emission of bright fluorescent signals solely in the existence of target molecules. This activatable aptamer-reporter, simultaneously monitoring various cellular molecules, thus provides higher selectively and specificity in CTC diagnosis [18].

## 2. Experimental Section

### 2.1. Cell Culture

The murine pancreatic cancer Panc02 was purchased from the American Type Culture Collection (ATCC, Manassas, VA, USA). Cells were cultured in Dulbecco’s modified eagle’s medium (DMEM, Thermo Scientific Hyclone, Waltham, MA, USA) supplemented with 10% fetal bovine serum (FBS, Gibco, Grand Island, NY, USA) and 1% penicillin/streptomycin (Gbico, Grand Island, NY, USA) at 5% CO_2_ and at 37 °C.

### 2.2. Preparation of an EpCAM Aptamer-Linked Molecular Beacon Conjugated with NIF Probes (NIF-EpCAM Aptamer-Linked Beacon and QD-Aptamer-Linked Beacon Probe)

To prepare the QD565 EpCAM ALB for in vitro experiment, we designed a single-stranded oligonucleotide with amine parts at the cohesive end, then linked with a beacon linker sequences. EpCAM ALB was slightly modified from that described in previous report [19]. The 3′-end with oligo-EpCAM binding sequence pairs (EpCAM ALB) were attached to the black hole quencher 2 (BHQ2; Bioneer, Inc., Daejeon, Korea). EpCAM/muc1 ALB oligonucleotides was synthesized and purchased from Bioneer (Daejeon, Korea). The entire EpCAM ALB was prepared as below: 63 nucleotide (n/t); amine-gggacacaatggacgtccgtag**ttctggctgactggtta**cccctctaacggccgacatgagag-BHQ2. The mutant oligonucleotide with sequence partially deleted sequences to the 5′-end of the short-sequence and a BHQ2 at the 3′-end was synthesized (41 n/t; amine-gggacacaatgg -acgtccgtagttctgcggccgacatgagag-BHQ2). Muc1 ALB was slightly modified from that described in previous report [20]. The entire muc1ALB was prepared as follows: 57 n/t; amine-aaccgcccaaatccctaagcttt**ggataccctggcaca**gacacactacacacgcaca-BHQ. QDs were purchased from Molecule Probe (Thermo Scientific Hyclone, Eugene, OR, USA). Underlined sequences mean target binding molecule of molecular beacon. Bold sequences indicate binding regions of targeting EpCAM or muc1 molecules. In vivo NIF-EpCAM ALB specific for the detection of EpCAM in mice were constructed with cy5.5 NIF probe instead of QD dye. According to the manufacturer’s method, the NH2-EpCAM ALB sequence was conjugated with the COOH-QD565 probe (designated as fluorophore dye) at a molar ratio of 2:1 in 0.1 M TE buffer (pH 7.0) with 78 mg/mL EDC (1-ethyl-3-(3-dimethylaminopropyl)carbodiimide hydrochloride) for 2 h at room temperature. To form with secondary structure for high binding ability of EpCAM ALB sequence, at the EpCAM target binding active site on the cell surface, 5 pmol QD_565_ EpCAM ALB or 50 pmol cy5.5 NIF EpCAM ALB was thermodynamically annealed at 94 °C and 72 °C PCR condition to construct a CTC cell-activatable EpCAM ALB sequences.

### 2.3. Analysis of the Construction of the EpCAM ALB and Muc1 ALB

Electrophoretic gel shift assay was performed to determine the construction of the EpCAM ALB or muc1 ALB using 1.5% agarose gel (1.5% agarose in nuclease-free 1X TAE buffer). Briefly, 5 pmol of the EpCAM ALB or muc1 ALB and QD_565_-EpCAM ALB or muc1 ALB were run on the gel. The conjugated pattern on agarose gel was further analyzed by EtBr dye using Gel Doc™ EZ Gel Documentation System (Bio-Rad, Hercules, CA, USA).

### 2.4. Specificity of the QD-EpCAM ALB for Sensing CTC

The quenched QD_565_-EpCAM ALB was incubated with various numbers of Panc02 cells (1 × 10^2^, 5 × 10^2^, 1 × 10^3^, 5 × 10^3^, 1 × 10^4^ and 5 × 10^4^ cells) to check the expression of extracellular EpCAM in a tube for 5 min at room temperature. EpCAM ALB was also able to bind to EpCAM on the surface of human cancer cells from different pathological origins, including MDA-MB-231 and Kato III. Furthermore, EpCAM ALB probe still provided the high selectivity against EpCAM-negative cell lines, including HEK-293T cells [20,21]. Compared to the parental sequence, the length of EpCAM ALB mutant is greatly reduced to 16 n/t long oligonucleotides, almost core sequence. Signal-on of the quenched QD_565_-EpCAM ALB was determined by fluorescence intensity and image.

### 2.5. Confocal Microscopy

Panc02 cells were seeded onto 35 mm cover glass-bottom dish (SPL Life Sciences Ltd., Pocheon, Korea), covered with a 13-mm diameter cover glass, and cultured for 24 h. Cells were treated with 2 pmol of the QD_565_-EpCAM ALB or 5 pmol of QD_525_-muc1 ALB probe. Following washes with PBS for 5 min two times, cells were fixed in 4% *p*-formaldehyde (Sigma-Aldrich, St. Louis, MO, USA). After washing three times with PBS for 5 min, mounting solution was applied. Image of cells were obtained by confocal laser scanning microscopy (Carl Zeiss LSM710, Weimer, Germany).

### 2.6. Detection of CTC in Mouse Bloods

To investigate the possibility of NIF-EpCAM ALB for CTC isolation from whole mouse blood samples (the volume of each blood samples was 10 μL) with caerulein was injected in Panc02-implanted mouse. Signal-on 5 pmol NIF-EpCAM ALB or 10 pmol muc1 ALB for one-step high affinity detection of CTCs in mouse whole blood samples were air-dried at room temperature for 5 min on a slide glass. Isolated mouse bloods were mixed with 5 pmol NIF-EpCAM ALB or 10 pmol NIF-muc1 ALB for 1 min at room temperature and were simultaneously added with H33342 (Invitrogen, Eugene, OR, USA) for nuclei stain. Then, images of cells covered with cover glass were obtained using LSM 710 microscopy (Carl Zeiss LSM710, Weimer, Germany).

### 2.7. In Vivo Near-Infrared Fluorescent Imaging of CTC Cells in Panc02-Implanted Mouse

All procedures involving animals, including housing and care, the method by which they were killed, and experimental protocols, were conducted accordance with a code of practice established by the Animal Care and Use Committee of Bundang Hospital of Seoul National University, Korea. This study was approved by the Seoul National University Bundang Hospital Institutional animal Care and Use Committee (BA1109-091/064-01). Eight-week-old female specific-pathogen-free C57/BL/6J mice with body weights of 20–22 g were obtained from Oriental Bio (Seongnam, Korea). They were maintained in plastic cages with sterilized paper bedding in a clean, air-conditioned room at 22 ± 2 °C. They are allowed to adapt to the new surrounding for 7 days with free access to a standard laboratory diet and water. The mice were caged in groups of 10 with free access to water. In the control group (*n* = 10), mice were anesthetized 4 weeks after Panc02 cell injection into the pancreatic tail. In the second group (*n* = 10), during 2 weeks after tumor injection, a suspension of 50 μg/kg of caerulein in PBS was injected intraperitoneally, then the mice were anesthetized 2 weeks after caerulein injection. In the normal group (*n* = 10, N group), the mice received no injection of cancer cells or caerulein. Anesthetized mice were imaged for NIRF fluorescence using a Peltier cooled charged-coupled device camera (Night OWL LB 983; Berthold Technologies, Bad Wildbad, Germany) to assess CTC existence at weeks 2 post-inoculation. The excitation source is a ring light used for illumination, mounted 12 cm above the mice. Filters of 671 nm and 707 nm were used to assess excitation and emission signals respectively. Using the WinLight 32 software (Berthold Technologies, Berthold, ND, USA), fluorescent signals (expressed in counts/s) from the images was calculated by selecting a rectangular region of interest around the tumor and integrating the signal of each pixel over the chosen area. To account for variations in auto-fluorescence over time and between mice, the rectangular region of interest was placed over an adjacent non-bone area to determine the background signal. This signal was then subtracted from the CTC signal. The tumor area was calculated using the Winlight32 software and expressed as mm^2^. The threshold of fluorescence emission was set to the level at which non-specific fluorescent signal was no longer detected in adjacent skin.

### 2.8. Statistical Analysis

All data are presented as the means ± standard deviation (SD). The significance of difference was evaluated using Student’s *t*-test (* *p* < 0.05 and ** *p* < 0.005).

## 3. Results

### 3.1. Characterization of the QD-EpCAM/muc1 ALB

**Figure 1:**
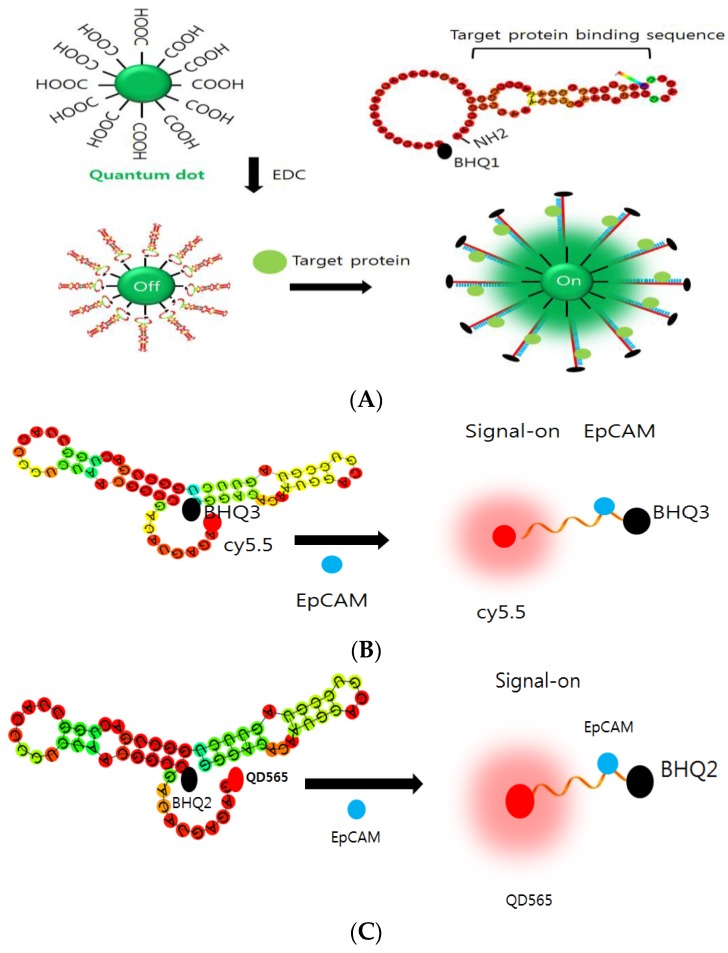
Schematic illustration of: QD_525_-muc1 ALB (**A**); cy5.5-EpCAM ALB (**B**); and QD_565_-EpCAM ALB (**C**). The EpCAM expression as an evidence of CTC was determined by the QD signal of ALB. The predicted secondary structures of full-length EpCAM ALB aptamer modeled using Mfold.

**Figure 2:**
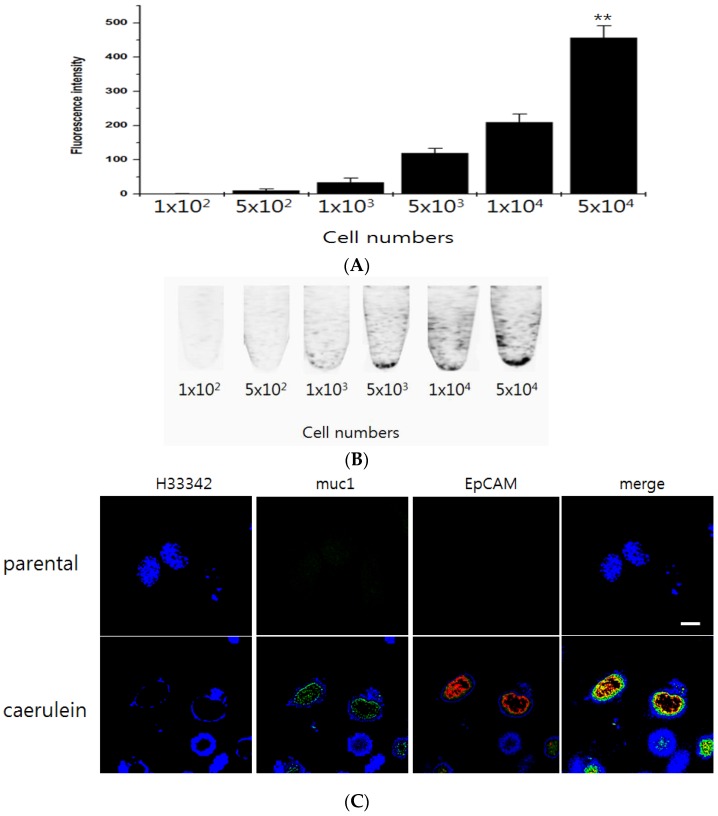
Specificity of the QD_565_-EpCAM ALB to sense EpCAM expression of CTCs in vitro: (**A**) LAS 4000 images of Panc02 cells treated with the QD_565_-EpCAM ALB in PCR tube were shown. A fixed concentration of the QDs was incubated with various cell numbers of the EpCAM-targeting protein to detect the optimal contents of EpCAM needed to perform the best specific effect; (**B**) Fluorescence intensity of the QD_565_-EpCAM ALB after incubated with the different cell numbers of Panc02 cells. ROI analysis from the fluorescence tube image showed that the fluorescence signal increased in a cells-dependent manner. Data are displayed as mean ± standard deviations of triplicate samples (** *p* < 0.005). The Confocal images QD525 and QD565 were recorded under excitation of 525 and 565 nm, respectively. The scale bar in the CLSM images is 20 μm. Fluorescence intensity of QD565-EpCAM/QD525-muc1 ALB on Panc02 cells after caerulein treatment (**C**).

In this study, a new monitoring method was developed with fluorophore-conjugated aptamer linked beacon (ALB) and used to image EpCAM or muc1 on the CTCs from the blood of mouse (Figure 1). ALB is synthesized with nucleic acid linked the quantum dot *(*QD) or Cy5.5 fluorescent dye (Figure 1C). The conjugation of QD was performed using electrophoretic shift method (Figure S2). The QD_565_-EpCAM ALB showed a shift in mobility when compared to the non-conjugated ALB. These results suggested that the shift is attributed to the QD conjugation (Figure S2). The integration of aptamer with the QD was checked by UV-spectrum (Figure S3). In this study, the EpCAM was chosen because it is highly expressed by pancreatic cancer and has a critical role in acute inflammation. For non-invasive imaging of EpCAM expression in CTC cells, the QD_565_-conjugated EpCAM ALB (QD_565_-EpCAM ALB) was designed. The QD_565_-EpCAM ALB consisted of the dual purpose sequences. First sequence is the single stranded oligonucleotide with aptamer sequence to the extracellular EpCAM of CTC cells. Second one is the oligonucleotide with a sequence partially fluorophore dye to the 5′-end. A black hole quencher molecule (BHQ) at the 3′-end of both probes was synthesized (Figure S4). Murine pancreatic cancer Panc02 cells were chosen as an EpCAM-nonexpressed cell lines that do not express extracellular EpCAM under parental cells. In the absence of EpCAM/muc1 expression in parental Panc02 cells, no fluorescence signal from the QD_565_-EpCAM/muc1 ALB were observed due to proximal quenching of QD_565_/QD_525_ (Figure 2C, upper panel). When EpCAM was expressed induced by caerulein treatment on Panc02 cells, fluorescence signal from extracellular membrane turned on via binding of EpCAM protein to EpCAM conformational affinity sequence selectively and detaching the quencher-sequence from the QD_565_-EpCAM ALB (Figure 2A,B).

### 3.2. Specificity of the QD-EpCAM ALB

To assay the specificity of the QD_565_-EpCAM ALB, EpCAM was gradually increased using dose-dependent increase treatment of caerulein on Panco2 cells. The fluorescence intensity of signal-on was attributed to specific binding of EpCAM protein to the EpCAM binding sequence of EpCAM ALB and sequential detachment of the quencher-sequence from the QD_565_-EpCAM ALB (Figure 2A). These cells were treated with 1 pmol of the QD_565_-EpCAM ALB and specificity was determined after 5 min. The result showed that without of caerulein treatment, QD_565_ fluorescence signal of the QD_565_-EpCAM ALB was quenched. However, the fluorescence signal solely increased in an extracellular EpCAM expression-dependent manner by caerulein treatment (Figure 2B).

### 3.3. Selectivity of QD-EpCAM ALB

To test whether our EpCAM aptamers are able to bind to EpCAM on the surface of human cancer cells from different pathological originals according to the previous report [21], we studied the interaction of EpCAM ALB aptamers with EpCAM-positive cell lines using flow cytometry and confocal analysis. Breast adenocarcinoma-derived cell line MDA-MB-231 and human gastric cancer cells line Kato III were chosen as positive cell lines, and human embryonic kidney cell line HEK-293T was chosen as the negative control.

**Figure 3:**
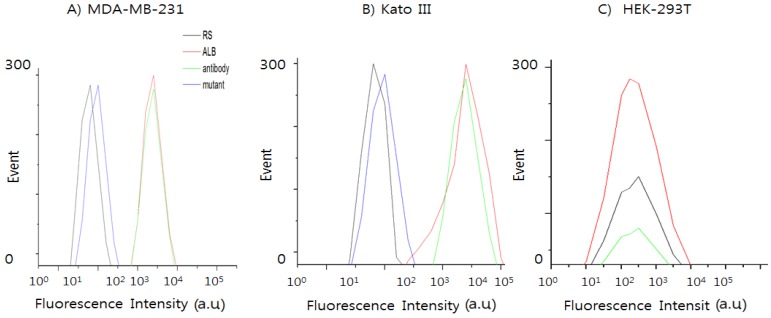
Selectivity study of various probe (RS; random sequence, EpCAM ALB, antibody, and mutant) against different cell lines including EpCAM-positive cell lines: (**A**) breast cancer cell line MDA-MB-231; (**B**) human gastric carcinoma cell line Kato III; and (**C**) negative cell line human kidney epithelial cell line HEK-293T.

EpCAM overexpression has been represented to be associated with a strongly invasive and aggressive cancer phenotype in breast cancer [22]. Therefore, to further confirm these data, the selective binding capability of the EpCAM ALB aptamer, antibody, random sequence and mutant against EpCAM-positive cancer cells and negative cancer cells were used to measure the percentage of expression in the MDA-MB-231 and Kato III cell lines (Figure 3A), and the percentage of positive signals obtained with the QD_565_-labeled ALB aptamer increased accordingly from 1.2% to 98.8% (Figure 3B). However, HEK-293T cells indicated 0% positive signals obtained with the QD_565_-labeled ALB aptamer (Figure 3C). As shown in Figure S5, EpCAM ALB aptamers were able to bind to the two different types of human cancer cell lines, but they did not bind to normal cell line (HEK-293T), indicating that the aptamers selected against recombinant EpCAM protein can also recognize the native EpCAM protein on live cell membranes (data not shown). The result suggests that the QD_565_-EpCAM ALB aptamer holds considerable potential for simple, rapid, and specific cancer cell detection in clinical samples. The binding ability of the ALB aptamer on QD_565_-EpCAM was found to be different from that of EpCAM antibody.

### 3.4. Imaging of Exogenous EpCAM/muc1 in Panc02 Cells

**Figure 4:**
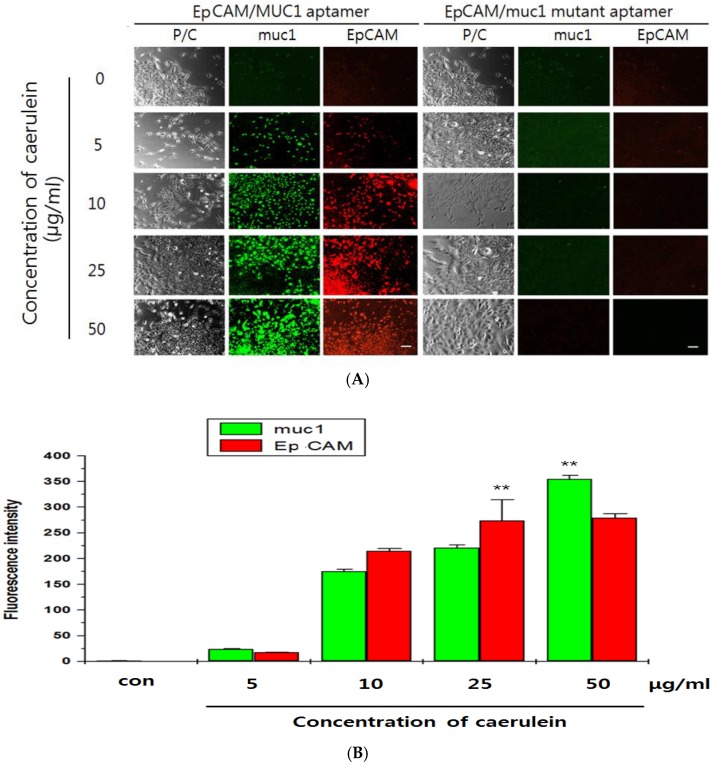
Confocal images of CTC existence from the Panc02 cells by the dose-dependent caerulein treatment after incubated with: EpCAM ALB (QD_565_, 1 pmol, specifically recognizes EpCAM), muc1-ALB (QD_525_-labeled, 2 pmol, specifically recognizes muc1) (**A**); and EpCAM/muc1 mutant probe (QD_525_/QD_565_-labeled, 2.5 pmol, specifically recognizes EpCAM/muc1, respectively) for 2 h (**B**). The Confocal images QD_525_ and QD_565_ were recorded under excitation of 525 and 565 nm, respectively. The scale bar in the CLSM images is 15 μm. Fluorescence intensity of QD-EpCAM/muc1 ALB on CTC cells after a dose-dependent caerulein. Data are displayed as mean ± standard deviations of triplicate samples (** *p* < 0.005). P/C: phase contrast.

Next, parental Panc02 cells, which express no EpCAM, were treated with various concentrations of caerulein with QD_565_-EpCAM ALB or QD_525_-muc1 ALB. Red fluorescence also increased in a fluorescence signal-on was attributed by extracellular EpCAM and green fluorescence similarly increased in a fluorescence signal-on for muc1 manner (Figure 4A,B). To confirm that the fluorescence signal-on was caused by extracellular EpCAM, 1 pmol of the QD_565_-EpCAM mutant ALB or 2 pmol of QD_525_-muc1 mutant ALB-treated Panc02 cells were further incubated with dose-dependent caerulein manner. Competitive affinity of the QD_565_-EpCAM and QD_565_-EpCAM mutant ALB or muc1 and QD_525_-muc1 mutant ALB to extracellular Ep-CAM or muc1 expression immediately disappeared to the fluorescence signal. Therefore, ALB was nontoxic to pancreatic cancer cells on functional targeting of EpCAM or muc1. The functionality of QD_565_-EpCAM ALB or QD_525_-muc1 ALB was determined during 5min of incubation (Figure 4B). The previous reports showed that VisuFect (VF)-conjugated beacon were nontoxic to mammalian cells and that the miRNA MB had no significant effect on functional targeting of miRNA [23]. Our QD_565_-EpCAM ALB/QD_525_-muc1 ALB designed in the current study similarly consisted of VF-MB system. Therefore, this indicates that the QD_565_-EpCAM ALB/QD_525_-muc1 ALB might not interrupt EpCAM expression in Panc02 cells. These results suggest that the signal-on of the QD_565_-EpCAM ALB or QD_525_-muc1 ALB was contributed by specific affinity of extracellular EpCAM or muc1 expression.

**Figure 5:**
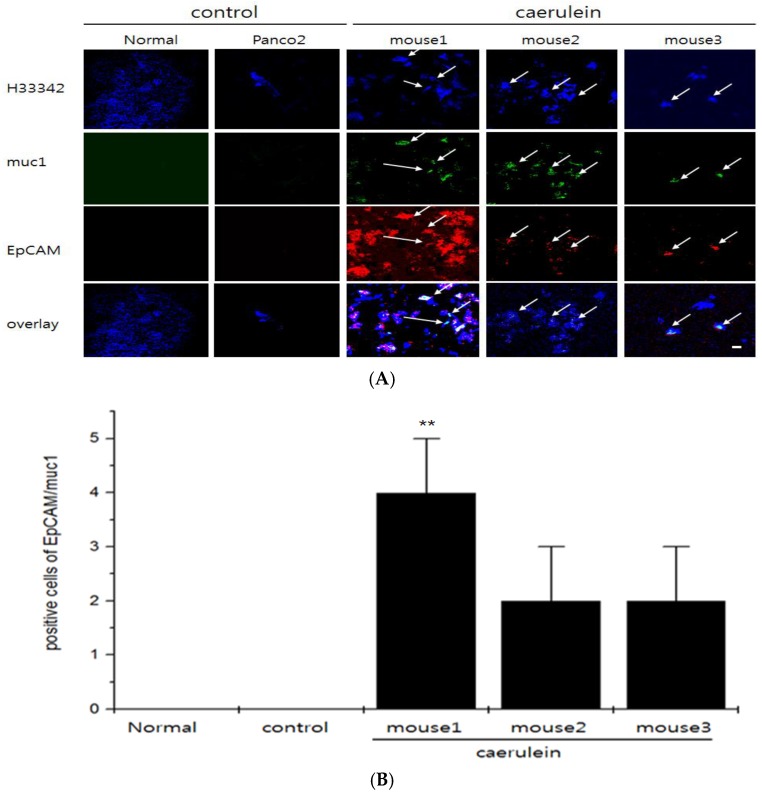
Confocal microscopy imaging of CTC cells mixed with bloods and NIF-EpCAM/muc1 ALB probes. Either 5 pmol NIF-EpCAM ALB or 10 pmol NIF-muc1 ALB was mixed with isolated-bloods two weeks after caerulein injection in Panc02-implanted mice (**A**). Fluorescence signals two weeks after caerulein treatment were significantly enhanced compared to those of the untreated caerulein in Panc02-implanted group. Positive cell numbers of QD-EpCAM/muc1 ALB on CTC cells in bloods after caerulein treatment (**B**). Data are represented as mean ± standard deviations of triplicate samples (** *p* < 0.005). Quantitative positive cells showed a higher fluorescent positive cells in blood with the caerulein-treated Panc02 cells than in mice without caerulein treatment (control) and normal mice, ** *p* < 0.005.

To compare the efficiency of NIF-EpCAM ALB and NIF-muc1 ALB on whole blood, only cy5.5 was conjugated to the 5′-end of the NIF-EpCAM ALB or NIF-muc1 ALB in the EpCAM or muc1 protein instead of QD. Resulting NIF-EpCAM ALB or NIF-muc1 ALB was mixed with mouse blood samples from Panc02-implanted mice in vitro. The result was shown that the fluorescence intensity of the NIF-EpCAM ALB or NIF-muc1 ALB was enhanced after caerulein treatment. NIF-ALB probe mixed normal and Panc02 without caerulein treatment did not show the fluorescence intensity due to the absence of EpCAM or muc1 expression of CTC existence (Figure 5, normal group and Panc02 group). Thus, the NIF-EpCAM ALB or NIF-muc1 ALB signal-on has high affinity and is efficient for CTC in mouse blood after caerulein treatment as inducer of acute inflammation (Figure 5, mouse1, mouse2, and mouse3). Moreover, the selective binding capability of QD_565_-EpCAM ALB to target cells in a human blood sample was further confirmed (Figure 5), suggesting the feasibility of using this aptamer in real blood samples.

### 3.5. In Vivo Imaging of Endogenous EpCAM/muc1of CTC during Metastasis of Panc02 Cells

**Figure 6:**
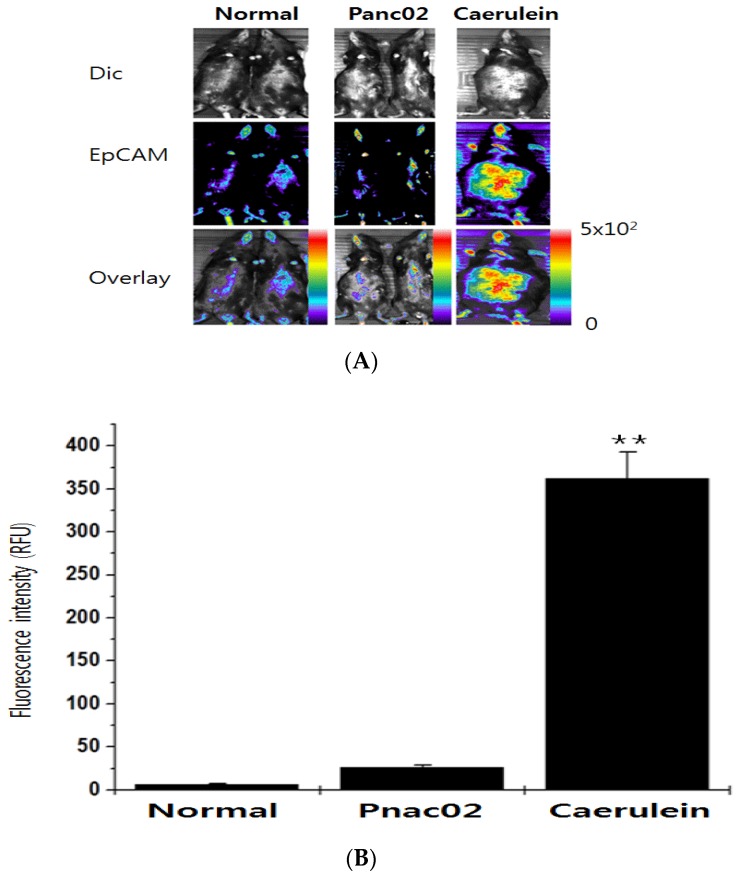
In vivo monitoring of the CTC existence pattern in mice with the NIF-EpCAM ALB incorporated into Panc02 cells. (**A**) The NIF-EpCAM ALB injection into mice was performed in the tail vein. The implanted trial of the Panc02 injection in pancreatic tissues, in the mice treated with caerulein for CTC existence, and placed by surgical implantation. An enhanced fluorescence signal in the CTC existence group (right) was detected compared to the group injected with phosphate-buffered saline (PBS) (left). Fluorescence images indicated that CTC cells of the implanted Panc02 cells in liver had similar observed; (**B**) Quantitative ROI analysis showed a higher fluorescence intensity in mice with the caerulein-treated Panc02 cells than in mice without caerulein treatment (middle), ** *p* < 0.005. Dic: differential interference contrast; RFU: relative fluorescence unit.

The sensitivity of the NIF-EpCAM ALB was further evaluated in mice that expressed high level of CTC by caerulein injection. The NIF-EpCAM ALB-intravenous injected mice were implanted with Panc02 cells and treated with caerulein. Interestingly, the CTC existence was observed with bright red fluorescence signals with cy5.5 with NIF-EpCAM ALB triggered by caerulein (Figure 6A, right row, Figure 6B). However, cy5.5 signals were not visualized in the control mice (Figure 6A, left and middle row). These results showed the sensitivity of EpCAM detection in mouse by the NIF-EpCAM ALB.

Optimum condition for EpCAM in vivo imaging using the NIF-EpCAM ALB for living mouse were determined as 1 × 10^7^ cells of Panc02 cells and 50 μg/kg caerulein injection for two weeks. Incubation of 30 min was needed to observe an EpCAM existence enhance in the cy5.5 fluorescence signal. In the case of 5 pmol of the NIF-EpCAM ALB, cy5.5 fluorescence signal was enhanced in mouse liver tissues after 30 min of treatment. It was noted that the fluorescence imaging of EpCAM can be visualized in the liver tissue. To confirm that the fluorescence signal-on was related to extracellular EpCAM expression, we synthesized another NIF-conjugated mutant EpCAM ALB to sense EpCAM as a negative control. The EpCAM is not detected in normal mouse liver regions by NIF-conjugated mutant EpCAM ALB but expressed by NIF-EpCAM ALB in the ICC analysis (data not shown).

The functionalities of the NIF-EpCAM ALB, such as specific quenching efficiency and the EpCAM specificity, were confirmed in test tubes and in Panc02 cells (Figure 2 and Figure 6). The NIF-EpCAM mutant ALB-treated mice showed no cy5.5 signal due to expression of EpCAM in Panc02-implanted mice by caerulein injection (Figure 4). These results verified that the signal-on of the NIF-EpCAM ALB was attributed by specific affinity of extracellular EpCAM expressed on the liver metastasis in the mouse. To confirm the fluorescence visualization of sensing EpCAM by the NIF-EpCAM ALB in the liver, an EpCAM ALB-monitoring probe was applied on the mouse. Most EpCAM and muc1 expression was solely monitored in blood and liver tissues of the Panc02-implanted mice by caerulein stimulation. These results are similar with many previous studies that have reported the presence of CTC [18]. We also evaluated the binding affinity of EpCAM in the blood samples of Panc02-implanted mice to confirm the EpCAM specificity of the NIF-EpCAM ALB monitoring probe after caerulein injection. As a result, EpCAM was detected within each blood samples, consistently with a previous report. These results suggested that the signal-on of the NIF-EpCAM ALB was performed by specific high affinity of extracellular EpCAM expressed both in mouse blood and liver tissues.

Bio imaging for CTC is clinically and industrially important because extracellular molecules in cancer cells have a significant effect on tumor progression and clinical diagnosis. Recent studies of diagnosis in clinical fields have focused on where these biomarkers are expressed in the CTC and their functions. However, there are few studies on live CTC bio imaging due to the technical limitations in the bio imaging of living CTC and in vivo. Since the EpCAM has been reported to be highly expressed in mouse CTC and important for metastatic progression, the NIF-conjugated EpCAM ALB can be useful for studying CTC functions in blood. This is the first report showing the noninvasive bio imaging of CTC existence in a living mouse and we hope that his simple, cost effective, and nontoxic method can be applied for bio imaging of biomarkers in CTC.

## 4. Discussion

In summary, we have designed a new monitor method for infinitesimal volumes of CTC-related proteins in bloods in slow speed and low CTC-detect yield. This assay can also discriminate the various CTC cells. The deliberated assay provides great potential in monitoring of biomarkers in cells or tissues and holds valuable information for biomedical research and clinical prognosis. In evidence to these previously studied methods for CTC monitoring, this assay has high detection specificity because it performs via detecting the recovered fluorescence signal, which is a result of the specific target-molecule-binding-triggered conformational change in the designed activatable aptamer beacon probe. In addition, this method can detect in complicated environments, very squalid conditions and at infinitesimal volumes.

## 5. Conclusions/Outlook

This study describes a method of QD-based fluorescence imaging CTC occurrence during cancer metastasis. For detect EpCAM/muc1 imaging, EpCAM/muc1-targeting oligonucleotides were linked to QDs. Frame of QDs was confirmed by fluorescence monitoring using exogenous and extracellular molecules. Imaging of exogenous EpCAM/muc1 expression was performed with NIF-EpCAM/muc1-ALB by caerulein triggering Panc02-implanted mouse. This QD-based signal-on technique can successfully be used for in vitro and in vivo monitoring and imaging of exogenous EpCAM/muc1.

## Supplementary Materials

The following are available online at http://www.mdpi.com/1424-8220/16/11/1909/s1, Figure S1: Schematic illustration of the NIF-EpCAM ALB. Multiple tumor-related proteins on CTC cell surface based on fluorescence enhancement induced by specific-affinity-triggered conformation alteration of the activated ALB probes, Figure S2: Gel electrophoresis of EpCAM ALB (lane1), muc-1 (lane2), QD_565_-conjugated EpCAM (lane3) and QD_525_-conjugated muc1 (lane4). Each sample was loaded on 1.5% agarose gel with a 100 bp molecular ladder. The conjugation pattern on gel electrophoresis was evaluated by UV excitation (235/345 nm), Figure S3: Fluorescence emission spectra of QD-muc1 (black line), QD-EpCAM (red line), and cy5.5-EpCAM (yellow green line). Excitation: 500 nm, scanning wavelength: 500–800 nm, with a band width of 25 nm, Figure S4: Schematic illustration of the design and function of the QD_565_-EpCAM ALB secondary structure by mFOLD, Figure S5: Confocal images of cultured MDA-MB-231, Kato III and HEK-293T cells labeled with the EpCAM/QD_565_ ALB. The final concentration of the EpCAM/QD_565_ ALB was 2 pM. Scale bars indicated 15 μm.
