# Supplementary material for: Optical Aptamer Probes of Fluorescent Imaging to Rapid Monitoring of Circulating Tumor Cell
Source: Sensors (Basel). 2016 Nov 23;16(11):1909. doi: 10.3390/s16111909 (PMC5134568; doi:10.3390/s16111909)
Supplement: Supplementary file 1 [file sensors-16-01909-s001.pdf]

Supplementary Materials: Optical Aptamer Probes of Fluorescent Imaging to Rapid Monitoring of Circulating Tumor Cell

Ji Yeon Hwang, Sang Tae Kim, Ho Seong Han, Kyunggon Kim and Jin Soo Han

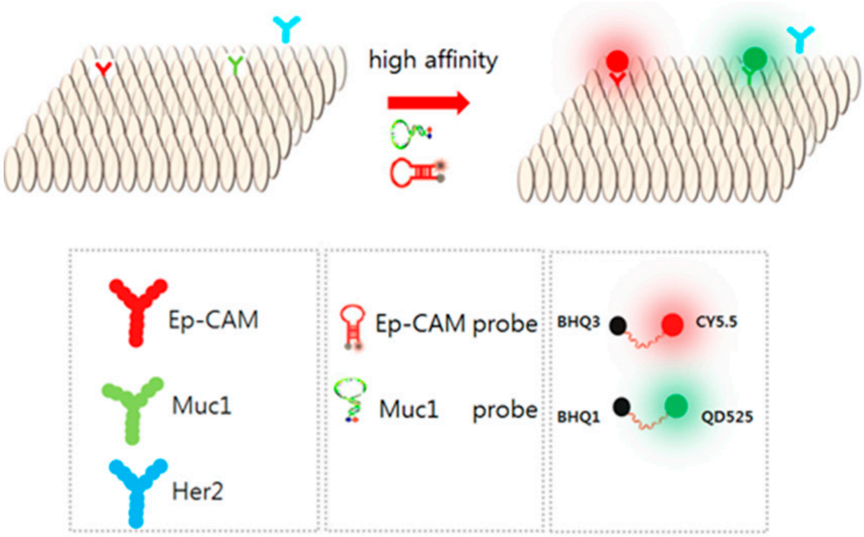

Figure S1. Schematic illustration of the NIF-EpCAM ALB. Multiple tumor-related proteins on CTC cell surface based on fluorescence enhancement induced by specific-affinity-triggered conformation alteration of the activated ALB probes.

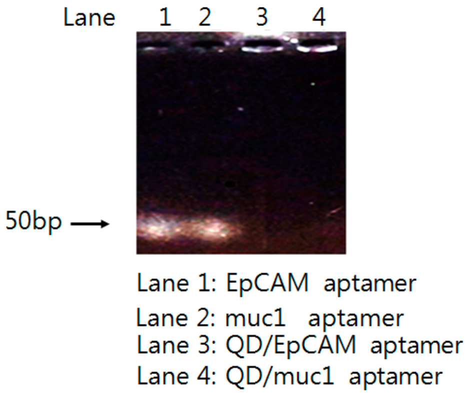

Figure S2. Gel electrophoresis of EpCAM ALB (lane1), muc-1 (lane2), QD_{565}-conjugated EpCAM (lane3) and QD_{525}-conjugated muc1 (lane4). Each sample was loaded on 1.5% agarose gel with a 100 bp molecular ladder. The conjugation pattern on gel electrophoresis was evaluated by UV excitation (235/345 nm).

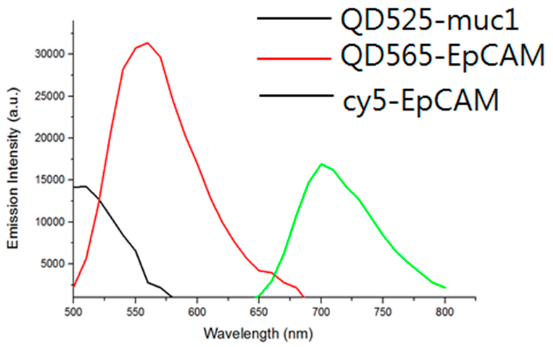

Figure S3. Fluorescence emission spectra of QD-muc1 (black line), QD-EpCAM (red line), and cy5.5-EpCAM (yellow green line). Excitation: 500 nm, scanning wavelength: 500–800 nm, with a band width of 25 nm.

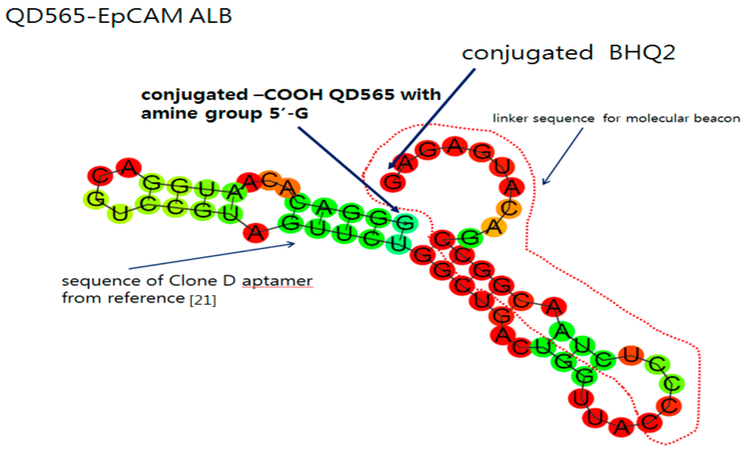

Figure S4. Schematic illustration of the design and function of the QD_{565}-EpCAM ALB secondary structure by mFOLD.

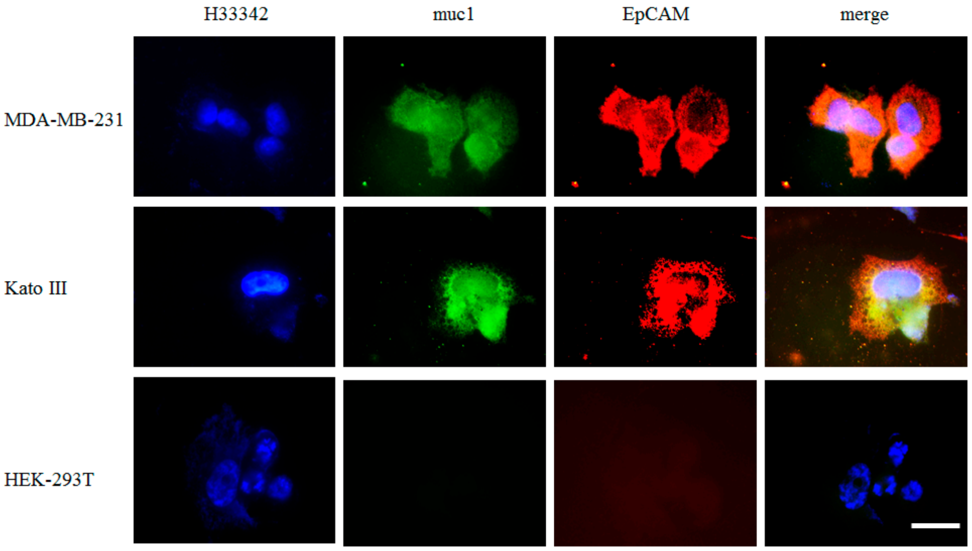

Figure S5. Confocal images of cultured MDA-MB-231, Kato III and HEK-293T cells labeled with the EpCAM/QD_{565} ALB. The final concentration of the EpCAM/QD_{565} ALB was 2 pM. Scale bars indicated 15 µm.
